# Supplementary material for: Concepts of psychosocial distress and help-seeking preferences among Indigenous adolescents: A qualitative study from Jharkhand, India
Source: PLOS Ment Health. 2026 May 4;3(5):e0000492. doi: 10.1371/journal.pmen.0000492 (PMC13138629; doi:10.1371/journal.pmen.0000492)
Supplement: S1 Text — (DOCX) [file pmen.0000492.s001.docx]

**S1 Text. Topic guides in English and Hindi**

**Topic guide for semi-structured interviews with adolescent boys and girls**

**Consent**

***Introductions***

Can you spend some time speaking to us?

First, I would like to learn a little about you:

- How old are you?
- How many people are in your family?
- What kind of work do they do?
- What does a normal weekday look like for you?

***Enjoyable activities***

- What are some of the activities you enjoy the most in your everyday life, for example at school, with your family or with your friends?
- Why do you like these so much?

***Challenges in daily life***

- Are there any times when you face worries or difficulties, for example:
  - At school? (probe: do you face any difficulties to continue your education, have you experienced any problems with peers or teachers at school?)
  - With your friends? (probe: can you share your joys and difficulties with them?)
  - In your family? (probe: can you support each other and discuss any problems?)
  - Are there other times where you face worries or difficulties? Why?

***Discussing a case story***

Thank you for sharing this. I am now going to share with you a short story about a young girl and ask you what you think is happening to her.

*Moni is 12 years old. This last month she has been crying about each and everything. If you say anything to her, she is likely to snap back at you. She gets really annoyed with her friends when they call her to play and asks them to leave her alone, so they don’t call her anymore. She used to have many interests, like playing games, helping with the housework, drawing. But now she’s just not interested in any of it. She just sits alone in the house, thinking and worrying. She doesn’t wake up for school unless her mother asks her several times to get out of bed. She’s stopped eating even her favourite foods, and she looks a lot thinner. She doesn’t have her usual energy anymore.*

- What do you think is happening to Moni?
- According to you why is she doing this/thinking this way?
- How would you explain what happened to her, in your own words?’ What are some of the other words you would use to describe someone like her who is feeling sad, thinking or worrying too much in a way that is different than normal? (Probe: what about in Ho – other languages spoken by the participant?)
  - Probe for each word – why did you choose this?
- Has this happened to you or to someone you know?
- Why do you think it happened?

- What did you/they do about it?

- In what situations do young people your age feel sad or very worried, like Moni?
- What do you think they do when this happens?

***Seeking help***

- In your opinion, what could be done to help young people who are feeling sad or are worrying too much, like Moni?
- If there was counselling / a talking therapy to help young people like Moni, who do you think would be trusted to give it? (Probe: a counsellor (AFHC), an older peer with training, someone else?)
- Where should they give it so that it reaches young people who need it? (Probe: school, clinic, community venue, any other place?)
- What are some of the things that would encourage young people to come for this help or stop them from coming? (probe: anything else?)
- What would you say to a friend to encourage them to go?

***Closing***

- Is there anything else that you want to say about what makes young people sad or worried, and what can be done to help them?
- Do you have any questions for me?

Thank you very much for giving us your time and your views.

**Topic guide for focus group discussions with adolescent boys and girls**

**(separate groups)**

***Introductions***

Thank you for agreeing to take part in this group discussion.

First, I would like to learn a little about each of you:

- How old are you?
- How many people are in your family?
- What kind of work do they do?
- What does a normal weekday look like for you?

***Experiences of being an adolescent***

- Earlier you were small children, now you are growing up. What has changed for you in your life since you’ve grown up? [Probe for: how you behave, physical appearance, food, friendships, the things that you are able to do and not do, relationship with parents, mobility, body changes, emotions, responsibilities, workload]
- What do you think are the problems faced by girls/boys your age in your community? [Probe for mobility, sports, employment, health, nutrition, alcohol, substance use, violence, early marriage, child trafficking]

***Discussing a case story***

Thank you for sharing this. I am now going to share with you a short story about a young girl and ask you what you think is happening to her.

*Moni is 12 years old. This last month she has been crying about each and everything. If you say anything to her, she is likely to snap back at you. She gets really annoyed with her friends when they call her to play and asks them to leave her alone, so they don’t call her anymore. She used to have many interests, like playing games, helping with the housework, drawing. But now she’s just not interested in any of it. She just sits alone in the house, thinking and worrying. She doesn’t wake up for school unless her mother asks her several times to get out of bed. She’s stopped eating even her favourite foods, and she looks a lot thinner. She doesn’t have her usual energy anymore.*

- What do you think is happening to Moni?
- According to you why is she doing this/thinking this way?
- How would you explain what happened to her, in your own words?’ What are some of the other words you would use to describe someone like her who is feeling sad, thinking or worrying too much in a way that is different than normal? (Probe: what about in Ho – other languages spoken by the participant?)
  - Which of these words are the easiest for young people to understand?

(Record top five words)

- - Probe for each top five words – why did you choose this word?
- In what situations do young people your age feel sad or very worried, like Moni? (Probe for examples)
- What do you think they do when this happens?

***Seeking help***

- In your opinion, what could be done to help young people who are feeling sad or are worrying too much, like Moni?
- If there was counselling / a talking therapy to help young people like Moni, who do you think would be trusted to give it? (Probe: a counsellor, an older peer with training, someone else?)
- Where should they give it so that it reaches young people who need it? (Probe: school, clinic, community venue, any other place?)

***Your role***

- What are some of the things that would encourage young people to come for this help or stop them from coming? (probe: anything else?)
- What would you say to a friend to encourage them to go?

***Closing***

- Is there anything else that you want to say about what makes young people sad or worried, and what can be done to help them?
- Do you have any questions for me?

Thank you very much for giving us your time and your views.

**Topic guide for focus group discussions with parents**

***Experiences of caring for adolescents***

What will you like to share with us as a parent of a adolescent? [Probe for: good experiences, challenging experiences]

- What do you think are the problems faced by the adolescents in your community? [Probe for: health, nutrition, employment, alcohol, substance use, violence, child trafficking]Do adolescents share their problems with you?
- How have you helped your child with these problems? [Probe for: what did you do, what was the problem, who else did you involve]
- In times of difficulty from whom and where does your child get advice and support? What are those difficulties? [Probe for: friends, siblings, family members, teachers, health workers, types of advice/support]
- Do you find out what is troubling your children when they are feeling low, how do you find out about it?

***Discussing a case story***

­­­­Thank you for sharing this. I am now going to share with you a short story about a young girl and ask you what you think is happening to her.

*Moni is 12 years old. This last month she has been crying about each and everything. If you say anything to her, she is likely to snap back at you. She gets really annoyed with her friends when they call her to play and asks them to leave her alone, so they don’t call her anymore. She used to have many interests, like playing games, helping with the housework, drawing. But now she’s just not interested in any of it. She just sits alone in the house, thinking and worrying. She doesn’t wake up for school unless her mother asks her several times to get out of bed. She’s stopped eating even her favourite foods, and she looks a lot thinner. She doesn’t have her usual energy anymore.*

- What do you think is happening to Moni?
- Why do you think this has happened to her?
- What are some of the other words you would use to describe someone like Moni who is feeling sad, thinking or worrying too much in a way that is different than normal? (Probe: what about in Ho – other languages spoken by participant?)
  - Which of these words are the easiest for young people to understand?

(Record top five words)

- - Probe for each top five words – why did you choose this word?

***Seeking help***

- In your opinion, what could be done to help young people who are feeling sad or are worrying too much, like Moni?
- If there was counselling / a talking therapy to help young people like Moni, who do you think would be trusted to give it? (Probe: a counsellor, an older peer with training, someone else?)
- Where should they give it so that it reaches young people who need it? (Probe: school, clinic, community venue, any other place?)
- What are some of the things that would encourage young people to come for this help or stop them from coming? (probe: anything else?)

***Your role***

- Would you encourage a young person to go for help?
- Would you have any concerns about this? What concerns?

***Closing***

- Is there anything else that you want to say about what makes young people sad or worried, and what can be done to help them?
- Do you have any questions for me?

Thank you very much for giving us your time and your views.

**Topic guide for semi-structured interviews with teachers**

***Experiences of working with adolescents***

- Can you tell me about your experiences of teaching adolescents? [Probe for: good experiences, challenging experiences]
- What are the common problems faced by the adolescents in your community? [Probe for: health, nutrition, employment, alcohol, substance use, violence, child trafficking]
- Who do adolescents go to for advice and support in times of difficulty? [Probe for: friends, siblings, family members, teachers, health workers]
- Do children come to you to discuss their problems? How have you helped adolescents to solve their problems? [Probe for: what did you do, what was the problem, who else was involved]

***Discussing a case story***

Thank you for sharing this. I am now going to share with you a short story about a young girl and ask you what you think is happening to her.

*Moni is 12 years old. This last month she has been crying about each and everything. If you say anything to her, she is likely to snap back at you. She gets really annoyed with her friends when they call her to play and asks them to leave her alone, so they don’t call her anymore. She used to have many interests, like playing games, helping with the housework, drawing. But now she’s just not interested in any of it. She just sits alone in the house, thinking and worrying. She doesn’t wake up for school unless her mother asks her several times to get out of bed. She’s stopped eating even her favourite foods, and she looks a lot thinner. She doesn’t have her usual energy anymore.*

- What do you think is happening to Moni?
- According to you why is she doing this/thinking this way?
- How would you explain what happened to her, in your own words?’ What are some of the other words you would use to describe someone like her who is feeling sad, thinking or worrying too much in a way that is different than normal? (Probe: what about in Ho – other languages spoken by the participant?)
  - Which of these words are the easiest for young people to understand?

(Record top five words)

- - Probe for each top five words – why did you choose this word?

***Seeking help***

- In your opinion, what could be done to help young people who are feeling sad or are worrying too much, like Moni?
- Have you every helped someone like Moni? What did you do?
- If there was counselling / a talking therapy to help young people like Moni, who do you think would be trusted to give it? (Probe: a counsellor, an older peer with training, someone else?)
- Where should they give it so that it reaches young people who need it? (Probe: school, clinic, community venue, any other place?)
- What are some of the things that would encourage young people to come for this help or stop them from coming? (probe: anything else?)

***Your role***

- What role could schools play in supporting this counselling/talking therapy?
- Why do you suggest this?

***Closing***

- Are there any other suggestions you would like to make about how to make this counselling / talking therapy work for young people?
- Do you have any questions for me?

Thank you very much for giving us your time and your views.

**Topic guide for semi-structured interviews**

**with frontline health workers**

**Working with the adolescents in their community**

- Can you tell me about your experiences of working with adolescents? [Probe for: girls, boys, unmarried, married]
- What are the main health problems of adolescents in your community? [Probe for: menstrual problems, anaemia, infections, undernutrition, pregnancy complications, mental health and violence, alcohol, substance use, injuries]
- What are your experiences of seeing adolescents with these problems? [Probe for: timing of seeking care, whether they come alone or along with their family, treatments provided, compliance]
- Can you share a particular example of a time where you provided care for an adolescent? [Probe for: the problem, support provided, what happened]
- How easy or difficult is it for you to work with adolescents compared to adults? [Probe for: compliance, availability, understanding, confidentiality, access, support provided]
- What are your experiences of providing adolescents (married and unmarried) with contraceptives? [Probe for: were you comfortable doing this, confidentiality, contraceptive type]

***Discussing a case story***

Thank you for sharing this. I am now going to share with you a short story about a young girl and ask you what you think is happening to her.

*Moni is 12 years old. This last month she has been crying about each and everything. If you say anything to her, she is likely to snap back at you. She gets really annoyed with her friends when they call her to play and asks them to leave her alone, so they don’t call her anymore. She used to have many interests, like playing games, helping with the housework, drawing. But now she’s just not interested in any of it. She just sits alone in the house, thinking and worrying. She doesn’t wake up for school unless her mother asks her several times to get out of bed. She’s stopped eating even her favourite foods, and she looks a lot thinner. She doesn’t have her usual energy anymore.*

- What do you think is happening to Moni?
- According to you why is she doing this/thinking this way?
- How would you explain what happened to her, in your own words?’ What are some of the other words you would use to describe someone like her who is feeling sad, thinking or worrying too much in a way that is different than normal? (Probe: what about in Ho – other languages spoken by the participant?)
  - Which of these words are the easiest for young people to understand?

(Record top five words)

- - Probe for each top five words – why did you choose this word?
- Have any young people come to you with these problems, or have you ever met a young person like that in the community?
- As a health worker, how do you see these things? What causes them?

***Seeking help***

- In your opinion, what could be done to help young people who are feeling sad or are worrying too much, like Moni?
- If there was counselling / a talking therapy to help young people like Moni, who do you think would be trusted to give it? (Probe: a counsellor, an older peer with training, a health worker, someone else?)
- Where should they give it so that it reaches young people who need it? (Probe: school, clinic, community venue, any other place?)
- What are some of the things that would encourage young people to come for this help or stop them from coming? (probe: anything else?)

***Your role***

- What role could health workers in supporting this counselling/talking therapy?
- What concerns would you have about this?

***Closing***

- Are there any other suggestions you would like to make about how to make this counselling / talking therapy work for young people?
- Do you have any questions for me? Thank you very much for giving us your time and your views.

किशोर लड़कों और लड़कियों के साथ अर्ध-संरचित साक्षात्कार के लिए विषय मार्गदर्शिका

अनुमति

परिचय

क्या आप हमसे बात करने में कुछ समय बिता सकते हैं?

सबसे पहले, मैं आपके बारे में थोड़ा जानना चाहूंगा:

- आपकी आयु कितनी है?

- आपके परिवार में कितने लोग हैं?

- वे किस तरह का काम करते हैं?

- आपके दैनिक दिन कैसे गुजर ता है?

**खुसी देने वाली गतिविधियाँ**

- ऐसी कौन सी गतिविधियाँ हैं जिनका आप अपने दैनिक जीवन में सबसे अधिक खुस लगता कर ने के लिए , जैसे कि स्कूल में, अपने परिवार के साथ या अपने दोस्तों के साथ?

-आप को ये इतना पसंद क्यों आता हैं? की

**दैनिक जीवन में चुनौतियाँ**

- क्या ऐसा कोई समय होता है जब आपको चिंता या दिकतों का सामना करना पड़ता है, जैसे कि:

स्कूल में? (जैसे कि: आपको अपनी शिक्षा जारी रखने में किसी दिकतों का सामना करना पड़ता है, क्या आपको स्कूल में साथियों या शिक्षकों के साथ किसी समस्या का अनुभव हुआ है?)

अपने दोस्तों के साथ? (जैसे कि: क्या आप अपनी खुशियाँ और दिकतों उनके साथ साझा कर सकते हैं?)

आपके परिवार में? (जैसे कि: क्या आप एक-दूसरे का सहयोग कर सकते हैं और किसी समस्या पर चर्चा कर सकते हैं?)

क्या ऐसे भी समय होता हैं जब आपको चिंता या दिकतों का सामना करना पड़ता है? क्यों?

**एक कहानी पर चर्चा**

इसे साझा करने के लिए आपको धन्यवाद। अब मैं आपके साथ एक युवा लड़की के बारे में एक छोटी कहानी साझा करने जा रहा हूं और आपसे पूछूंगा कि आपको क्या लगता है कि उसके साथ क्या हो रहा है।

मोनी 12 साल की है. पिछले महीने से वह हर बात पर रो रही है। यदि आप उससे कुछ भी कहते हैं, तो संभव है कि वह आप को उल्टा जबाब देगी । जब उसके दोस्त उसे खेलने के लिए बुलाते हैं तो वह उससे बहुत नाराज़ हो जाती है और उनसे उसे अकेले छोड़ने के लिए कहती है, इसलिए वे उसे अब नहीं बुलाते हैं। उसकी कई रुचियां थीं, जैसे गेम खेलना, घर के काम में मदद करना, चित्र बनाना। लेकिन अब उसे इस सब मे कोई रूचि नहीं है। वह घर में अकेली बैठी सोचती और चिंता करती रहती है। वह तब तक स्कूल के लिए नहीं उठती जब तक उसकी माँ उसे बिस्तर से उठने के लिए कई बार नहीं कहती। उसने अपना पसंदीदा खाना भी खाना बंद कर दिया है और वह बहुत पतली दिखती है। अब उसमें पहले जैसे पूर्ती नहीं रही।

- आपको क्या लगता है मोनी के साथ क्या हो रहा है?

- आपके अनुसार वह ऐसा क्यों कर रही है/ऐसा क्यों सोच रही है?

- उसके साथ जो हुआ, उसे आप अपने शब्दों में कैसे समझाएंगे?' उसके जैसे किसी व्यक्ति का वर्णन करने के लिए आप किन अन्य शब्दों का उपयोग करेंगे, जो सामान्य से अलग तरीके से दुखी, सोच या बहुत अधिक चिंता कर रहा है? (जैसे कि: ‘हो’ में - प्रतिभागी द्वारा बोली जाने वाली अन्य भाषाओं के बारे में क्या?)

प्रत्येक शब्द की बारे मैं बिस्तार से जाने कि कोशिश करें - आपने इसे क्यों चुना?

- क्या आपके साथ या आपके किसी जानने वाले के साथ ऐसा हुआ है?

- आपको क्या लगता है ऐसा क्यों हुआ?

- आपने/ उनके परिवार वालो ने इसके बारे में क्या किया?

• मोनी की तरह आपकी उम्र के युवा किन स्थितियों में दुखी या बहुत चिंतित महसूस करते हैं?

• आपको क्या लगता है कि ऐसा होने पर वे क्या करते हैं?

**मदद मांगना**

• आपकी राय में, मोनी जैसे उन युवाओं की मदद के लिए क्या किया जा सकता है जो दुःख महसूस कर रहे हैं या बहुत ज्यादा चिंता कर रहे हैं?

• अगर मोनी जैसे युवाओं की मदद के लिए परामर्श /बातचीत कि सुबिधा होती, तो आपको क्या लगता है ये सुबिधा देने के लिए किस पर भरोसा किया जाएगा? (जैसे कि: एक परामर्शदाता (एए फ एचसी), प्रशिक्षण प्राप्त एक पुराना सहकर्मी, कोई और?)

• उक्त सेवा कहां देना चाहिए ताकि यह उन युवाओं तक पहुंच सके जिनको इसकी आवश्यकता है? (जैसे कि: स्कूल, क्लिनिक, सामुदायिक स्थल, कोई अन्य स्थान?)

• ऐसी कौन सी चीजें हैं जो युवाओं को इस मदद के लिए आने के लिए प्रोत्साहित करेंगी या उन्हें आने से रोकेंगी? (जैसे कि: कुछ और?)

• आप किसी मित्र को सेवा जाने के लिए तक कैसे प्रोत्साहित कर के क्या कहेंगे?

**समापन**

• क्या आप कुछ और कहना चाहते हैं जिसके बारे में युवा लोग दुखी या चिंतित हैं, और उनकी मदद के लिए क्या किया जा सकता है?

• क्या आपके पास मेरे लिए कोई सवाल है?

हमें अपना समय और अपने विचार देने के लिए बहुत-बहुत धन्यवाद।

किशोर लड़कों और लड़कियों के साथ फोकस समूह चर्चा के लिए विषय मार्गदर्शिका

(अलग समूह)

परिचय

इस समूह चर्चा में भाग लेने और सहमती के लिए धन्यवाद।

सबसे पहले, मैं आप में से प्रत्येक के बारे में थोड़ा जानना चाहूंगा:

- आपकी आयु कितनी है?

- आपके परिवार में कितने लोग हैं?

- वे किस तरह का काम करते हैं?

- आपके लिए सामान्य कार्यदिवस कैसा दिखता है?

किशोर होने के अनुभव

• पहले आप छोटे बच्चे थे, अब बड़े हो रहे हो। जब से आप बड़े हुए हैं आपके जीवन में क्या बदलाव आया है? [जैसे कि: आप कैसा व्यवहार करते हैं, शारीरिक रूप, भोजन, दोस्ती, कौन सी चीजें आप करने में सक्षम हैं और क्या नहीं, माता-पिता के साथ संबंध, आने जाने , शारीरिक परिवर्तन, भावनाएं, जिम्मेदारियां, कार्यभार]

• आपके अनुसार आपके समुदाय में आपकी उम्र की लड़कियों/लड़कों को किन समस्याओं का सामना करना पड़ता है? [आने जाने, खेल, रोजगार, स्वास्थ्य, पोषण, शराब, मादक द्रव्यों का उपयोग, हिंसा, कम उम्र में विवाह, बाल तस्करी ]

**एक कहानी पर चर्चा**

इसे साझा करने के लिए आपको धन्यवाद। अब मैं आपके साथ एक युवा लड़की के बारे में एक छोटी कहानी साझा करने जा रहा हूं और आपसे पूछूंगा कि आपको क्या लगता है कि उसके साथ क्या हो रहा है।

मोनी 12 साल की है. पिछले महीने से वह हर बात पर रो रही है। यदि आप उससे कुछ भी कहते हैं, तो संभव है कि वह आप को उल्टा जबाब देगी । जब उसके दोस्त उसे खेलने के लिए बुलाते हैं तो वह उससे बहुत नाराज़ हो जाती है और उनसे उसे अकेले छोड़ने के लिए कहती है, इसलिए वे उसे अब नहीं बुलाते हैं। उसकी कई रुचियां थीं, जैसे गेम खेलना, घर के काम में मदद करना, चित्र बनाना। लेकिन अब उसे इस सब मे कोई रूचि नहीं है। वह घर में अकेली बैठी सोचती और चिंता करती रहती है। वह तब तक स्कूल के लिए नहीं उठती जब तक उसकी माँ उसे बिस्तर से उठने के लिए कई बार नहीं कहती। उसने अपना पसंदीदा खाना भी खाना बंद कर दिया है और वह बहुत पतली दिखती है। अब उसमें पहले जैसे पूर्ती नहीं रही।

- आपको क्या लगता है मोनी के साथ क्या हो रहा है?

- आपके अनुसार वह ऐसा क्यों कर रही है/ऐसा क्यों सोच रही है?

- उसके साथ जो हुआ, उसे आप अपने शब्दों में कैसे समझाएंगे?' उसके जैसे किसी व्यक्ति का वर्णन करने के लिए आप किन अन्य शब्दों का उपयोग करेंगे, जो सामान्य से अलग तरीके से दुखी, सोच या बहुत अधिक चिंता कर रहा है? (जैसे कि: ‘हो’ में - प्रतिभागी द्वारा बोली जाने वाली अन्य भाषाओं के बारे में क्या?)

o प्रत्येक शब्द की बारे मैं बिस्तार से जाने कि कोशिश करें - आपने इसे क्यों चुना?

- क्या आपके साथ या आपके किसी जानने वाले के साथ ऐसा हुआ है?

- आपको क्या लगता है ऐसा क्यों हुआ?

- आपने/ उनके परिवार वालो ने इसके बारे में क्या किया?

• मोनी की तरह आपकी उम्र के युवा किन स्थितियों में दुखी या बहुत चिंतित महसूस करते हैं?

• आपको क्या लगता है कि ऐसा होने पर वे क्या करते हैं?

**मदद मांगना**

• आपकी राय में, मोनी जैसे उन युवाओं की मदद के लिए क्या किया जा सकता है जो दुःख महसूस कर रहे हैं या बहुत ज्यादा चिंता कर रहे हैं?

• अगर मोनी जैसे युवाओं की मदद के लिए परामर्श /बातचीत कि सुबिधा होती, तो आपको क्या लगता है ये सुबिधा देने के लिए किस पर भरोसा किया जाएगा? (जांच: एक परामर्शदाता (एए फ एचसी), प्रशिक्षण प्राप्त एक पुराना सहकर्मी, कोई और?)

• उक्त सेवा कहां देना चाहिए ताकि यह उन युवाओं तक पहुंच सके जिनको इसकी आवश्यकता है? (जैसे कि: स्कूल, क्लिनिक, सामुदायिक स्थल, कोई अन्य स्थान?)

• ऐसी कौन सी चीजें हैं जो युवाओं को इस मदद के लिए आने के लिए प्रोत्साहित करेंगी या उन्हें आने से रोकेंगी? (जैसे कि :कुछ और?)

• आप किसी मित्र को सेवा जाने के लिए तक कैसे प्रोत्साहित कर के क्या कहेंगे?

**समापन**

• क्या आप कुछ और कहना चाहते हैं जिसके बारे में युवा लोग दुखी या चिंतित हैं, और उनकी मदद के लिए क्या किया जा सकता है?

• क्या आपके पास मेरे लिए कोई सवाल है?

हमें अपना समय और अपने विचार देने के लिए बहुत-बहुत धन्यवाद।

माता-पिता के साथ फोकस समूह चर्चा के लिए विषय मार्गदर्शिका

किशोरों की देखभाल के अनुभव

• एक किशोर के माता-पिता के रूप में आप हमारे साथ क्या साझा करना चाहेंगे? [जांच: अच्छे अनुभव, चुनौतीपूर्ण अनुभव]

• आपके अनुसार आपके समुदाय में किशोरों को किन समस्याओं का सामना करना पड़ता है? [जांच: स्वास्थ्य, पोषण, रोजगार, शराब, मादक द्रव्यों का सेवन, हिंसा, बाल तस्करी] क्या किशोर अपनी समस्याएं आपके साथ साझा करते हैं?

• आपने अपने बच्चे को इन समस्याओं से निपटने में कैसे मदद की है? [इसकी जांच: आपने क्या किया, समस्या क्या थी, आपने और किसे शामिल किया]

• कठिनाई के समय आपके बच्चे को किससे और कहाँ से सलाह और सहायता मिलती है? वे कठिनाइयाँ क्या हैं? [इनके लिए जांच: मित्र, भाई-बहन, परिवार के सदस्य, शिक्षक, स्वास्थ्य कार्यकर्ता, सलाह/सहायता के प्रकार]

• क्या आप पता लगाते हैं कि जब आपके बच्चे उदास महसूस करते हैं तो उन्हें क्या परेशानी हो रही है, आप इसके बारे में कैसे पता लगाएंगे?

**एक मामले की कहानी पर चर्चा**

इसे साझा करने के लिए धन्यवाद। अब मैं आपके साथ एक युवा लड़की के बारे में एक छोटी कहानी साझा करने जा रहा हूं और आपसे पूछूंगा कि आपको क्या लगता है कि उसके साथ क्या हो रहा है।

मोनी 12 साल की है. पिछले महीने से वह हर बात पर रो रही है। यदि आप उससे कुछ भी कहते हैं, तो संभव है कि वह आप को उल्टा जबाब देगी । जब उसके दोस्त उसे खेलने के लिए बुलाते हैं तो वह उससे बहुत नाराज़ हो जाती है और उनसे उसे अकेले छोड़ने के लिए कहती है, इसलिए वे उसे अब नहीं बुलाते हैं। उसकी कई रुचियां थीं, जैसे गेम खेलना, घर के काम में मदद करना, चित्र बनाना। लेकिन अब उसे इसमें कोई रूचि नहीं है। वह घर में अकेली बैठी सोचती और चिंता करती रहती है। वह तब तक स्कूल के लिए नहीं उठती जब तक उसकी माँ उसे बिस्तर से उठने के लिए कई बार नहीं कहती। उसने अपना पसंदीदा खाना भी खाना बंद कर दिया है और वह बहुत पतली दिखती है। अब उसमें पहले जैसे पूर्ती नहीं रही।

- आपको क्या लगता है मोनी के साथ क्या हो रहा है?

- आपके अनुसार वह ऐसा क्यों कर रही है/ऐसा क्यों सोच रही है?

- उसके साथ जो हुआ, उसे आप अपने शब्दों में कैसे समझाएंगे?' उसके जैसे किसी व्यक्ति का वर्णन करने के लिए आप किन अन्य शब्दों का उपयोग करेंगे, जो सामान्य से अलग तरीके से दुखी, सोच या बहुत अधिक चिंता कर रहा है? (जांच: हो में - प्रतिभागी द्वारा बोली जाने वाली अन्य भाषाओं के बारे में क्या?)

o प्रत्येक शब्द की जाँच करें - आपने इसे क्यों चुना?

- क्या आपके साथ या आपके किसी जानने वाले के साथ ऐसा हुआ है?

- आपको क्या लगता है ऐसा क्यों हुआ?

- आपने/उन्होंने इसके बारे में क्या किया?

• मोनी की तरह आपकी उम्र के युवा किन स्थितियों में दुखी या बहुत चिंतित महसूस करते हैं?

• आपको क्या लगता है कि ऐसा होने पर वे क्या करते हैं?

**मदद मांगना**

• आपकी राय में, मोनी जैसे उन युवाओं की मदद के लिए क्या किया जा सकता है जो दुःख महसूस कर रहे हैं या बहुत ज्यादा चिंता कर रहे हैं?

• अगर मोनी जैसे युवाओं की मदद के लिए परामर्श /बातचीत होती, तो आपको क्या लगता है ये सुबिधा देने के लिए किस पर भरोसा किया जाएगा? (जांच: एक परामर्शदाता (एए फ एचसी), प्रशिक्षण प्राप्त एक पुराना सहकर्मी, कोई और?)

• उक्त सेवा कहां देना चाहिए ताकि यह उन युवाओं तक पहुंच सके जिनको इसकी आवश्यकता है? (जांच: स्कूल, क्लिनिक, सामुदायिक स्थल, कोई अन्य स्थान?)

• ऐसी कौन सी चीजें हैं जो युवाओं को इस मदद के लिए आने के लिए प्रोत्साहित करेंगी या उन्हें आने से रोकेंगी? (जांच: कुछ और?)

• आप किसी मित्र को सेवा जाने के लिए तक कैसे प्रोत्साहित कर के क्या कहेंगे?

**समापन**

• क्या आप कुछ और कहना चाहते हैं जिसके बारे में युवा लोग दुखी या चिंतित हैं, और उनकी मदद के लिए क्या किया जा सकता है?

• क्या आपके पास मेरे लिए कोई सवाल है?

हमें अपना समय और अपने विचार देने के लिए बहुत-बहुत धन्यवाद।

शिक्षकों के साथ अर्ध-संरचित साक्षात्कार के लिए विषय मार्गदर्शिका

किशोरों के साथ काम करने का अनुभव

• क्या आप मुझे किशोरों को पढ़ाने के अपने अनुभवों के बारे में बता सकते हैं? [जांच: अच्छे अनुभव, चुनौतीपूर्ण अनुभव]

• आपके समुदाय में किशोरों को किन सामान्य समस्याओं का सामना करना पड़ता है? [इनकी जांच: स्वास्थ्य, पोषण, रोजगार, शराब, मादक द्रव्यों का उपयोग, हिंसा, बाल तस्करी]

• कठिनाई के समय किशोर सलाह और सहायता के लिए किसके पास जाते हैं? [इनकी जांच: दोस्त, भाई-बहन, परिवार के सदस्य, शिक्षक, स्वास्थ्य कार्यकर्ता]

• क्या बच्चे आपके पास अपनी समस्याओं पर चर्चा करने आते हैं? आपने किशोरों को उनकी समस्याओं को हल करने में कैसे मदद की है? [इसकी जांच: आपने क्या किया, समस्या क्या थी, और कौन शामिल था]

एक मामले की कहानी पर चर्चा

इसे साझा करने के लिए आपको धन्यवाद। अब मैं आपके साथ एक युवा लड़की के बारे में एक छोटी कहानी साझा करने जा रहा हूं और आपसे पूछूंगा कि आपको क्या लगता है कि उसके साथ क्या हो रहा है।

मोनी 12 साल की है. पिछले महीने से वह हर बात पर रो रही है। यदि आप उससे कुछ भी कहते हैं, तो संभव है कि वह आप को उल्टा जबाब देगी । जब उसके दोस्त उसे खेलने के लिए बुलाते हैं तो वह उससे बहुत नाराज़ हो जाती है और उनसे उसे अकेले छोड़ने के लिए कहती है, इसलिए वे उसे अब नहीं बुलाते हैं। उसकी कई रुचियां थीं, जैसे गेम खेलना, घर के काम में मदद करना, चित्र बनाना। लेकिन अब उसे इसमें कोई रूचि नहीं है। वह घर में अकेली बैठी सोचती और चिंता करती रहती है। वह तब तक स्कूल के लिए नहीं उठती जब तक उसकी माँ उसे बिस्तर से उठने के लिए कई बार नहीं कहती। उसने अपना पसंदीदा खाना भी खाना बंद कर दिया है और वह बहुत पतली दिखती है। अब उसमें पहले जैसे पूर्ती नहीं रही।

- आपको क्या लगता है मोनी के साथ क्या हो रहा है?

- आपके अनुसार वह ऐसा क्यों कर रही है/ऐसा क्यों सोच रही है?

- उसके साथ जो हुआ, उसे आप अपने शब्दों में कैसे समझाएंगे?' उसके जैसे किसी व्यक्ति का वर्णन करने के लिए आप किन अन्य शब्दों का उपयोग करेंगे, जो सामान्य से अलग तरीके से दुखी, सोच या बहुत अधिक चिंता कर रहा है? (जांच: हो में - प्रतिभागी द्वारा बोली जाने वाली अन्य भाषाओं के बारे में क्या?)

o प्रत्येक शब्द की जाँच करें - आपने इसे क्यों चुना?

- क्या आपके साथ या आपके किसी जानने वाले के साथ ऐसा हुआ है?

- आपको क्या लगता है ऐसा क्यों हुआ?

- आपने/उन्होंने इसके बारे में क्या किया?

• मोनी की तरह आपकी उम्र के युवा किन स्थितियों में दुखी या बहुत चिंतित महसूस करते हैं?

• आपको क्या लगता है कि ऐसा होने पर वे क्या करते हैं?

**मदद मांगना**

• आपकी राय में, मोनी जैसे उन युवाओं की मदद के लिए क्या किया जा सकता है जो दुःख महसूस कर रहे हैं या बहुत ज्यादा चिंता कर रहे हैं?

• अगर मोनी जैसे युवाओं की मदद के लिए परामर्श /बातचीत होती, तो आपको क्या लगता है ये सुबिधा देने के लिए किस पर भरोसा किया जाएगा? (जांच: एक परामर्शदाता (एए फ एचसी), प्रशिक्षण प्राप्त एक पुराना सहकर्मी, कोई और?)

• उक्त सेवा कहां देना चाहिए ताकि यह उन युवाओं तक पहुंच सके जिनको इसकी आवश्यकता है? (जांच: स्कूल, क्लिनिक, सामुदायिक स्थल, कोई अन्य स्थान?)

• ऐसी कौन सी चीजें हैं जो युवाओं को इस मदद के लिए आने के लिए प्रोत्साहित करेंगी या उन्हें आने से रोकेंगी? (जांच: कुछ और?)

• आप किसी मित्र को सेवा जाने के लिए तक कैसे प्रोत्साहित कर के क्या कहेंगे?

**समापन**

• क्या आप कुछ और कहना चाहते हैं जिसके बारे में युवा लोग दुखी या चिंतित हैं, और उनकी मदद के लिए क्या किया जा सकता है?

• क्या आपके पास मेरे लिए कोई सवाल है?

हमें अपना समय और अपने विचार देने के लिए बहुत-बहुत धन्यवाद।

अर्ध-संरचित साक्षात्कार के लिए विषय मार्गदर्शिका

**अग्रिम पंक्ति के स्वास्थ्य कार्यकर्ताओं के साथ**

अपने समुदाय में किशोरों के साथ काम करना

• क्या आप मुझे किशोरों के साथ काम करने के अपने अनुभवों के बारे में बता सकते हैं? [जांच: लड़कियों, लड़कों, अविवाहित, विवाहित]

• आपके समुदाय में किशोरों की मुख्य स्वास्थ्य समस्याएं क्या हैं? [इनकी जांच: मासिक धर्म संबंधी समस्याएं, एनीमिया, संक्रमण, अल्पपोषण, गर्भावस्था संबंधी जटिलताएं, मानसिक स्वास्थ्य और हिंसा, शराब, मादक द्रव्यों का सेवन, चोटें]

• किशोरों को इन समस्याओं से ग्रस्त देखकर आपके क्या अनुभव हैं? [इनकी जांच: देखभाल लेने का समय, चाहे वे अकेले आएं या अपने परिवार के साथ, उपलब्ध कराए गए उपचार, अनुपालन]

• क्या आप उस समय का कोई विशेष उदाहरण साझा कर सकते हैं जब आपने किसी किशोर की देखभाल की हो? [इनकी जांच: समस्या, सहायता प्रदान की गई, क्या हुआ]

• वयस्कों की तुलना में किशोरों के साथ काम करना आपके लिए कितना आसान या कठिन है? [इनकी जांच: अनुपालन, उपलब्धता, समझ, गोपनीयता, पहुंच, प्रदान की गई सहायता]

• किशोरों (विवाहित और अविवाहित) को गर्भनिरोधक उपलब्ध कराने के आपके अनुभव क्या हैं? [इसके लिए जांच: क्या आप ऐसा करने में सहज थे, गोपनीयता, गर्भनिरोधक प्रकार]

एक मामले की कहानी पर चर्चा

इसे साझा करने के लिए आपको धन्यवाद। अब मैं आपके साथ एक युवा लड़की के बारे में एक छोटी कहानी साझा करने जा रहा हूं और आपसे पूछूंगा कि आपको क्या लगता है कि उसके साथ क्या हो रहा है।

मोनी 12 साल की है. पिछले महीने से वह हर बात पर रो रही है। यदि आप उससे कुछ भी कहते हैं, तो संभव है कि वह आप को उल्टा जबाब देगी । जब उसके दोस्त उसे खेलने के लिए बुलाते हैं तो वह उससे बहुत नाराज़ हो जाती है और उनसे उसे अकेले छोड़ने के लिए कहती है, इसलिए वे उसे अब नहीं बुलाते हैं। उसकी कई रुचियां थीं, जैसे गेम खेलना, घर के काम में मदद करना, चित्र बनाना। लेकिन अब उसे इसमें कोई रूचि नहीं है। वह घर में अकेली बैठी सोचती और चिंता करती रहती है। वह तब तक स्कूल के लिए नहीं उठती जब तक उसकी माँ उसे बिस्तर से उठने के लिए कई बार नहीं कहती। उसने अपना पसंदीदा खाना भी खाना बंद कर दिया है और वह बहुत पतली दिखती है। अब उसमें पहले जैसे पूर्ती नहीं रही।

- आपको क्या लगता है मोनी के साथ क्या हो रहा है?

- आपके अनुसार वह ऐसा क्यों कर रही है/ऐसा क्यों सोच रही है?

- उसके साथ जो हुआ, उसे आप अपने शब्दों में कैसे समझाएंगे?' उसके जैसे किसी व्यक्ति का वर्णन करने के लिए आप किन अन्य शब्दों का उपयोग करेंगे, जो सामान्य से अलग तरीके से दुखी, सोच या बहुत अधिक चिंता कर रहा है? (जांच: हो में - प्रतिभागी द्वारा बोली जाने वाली अन्य भाषाओं के बारे में क्या?)

o प्रत्येक शब्द की जाँच करें - आपने इसे क्यों चुना?

- क्या आपके साथ या आपके किसी जानने वाले के साथ ऐसा हुआ है?

- आपको क्या लगता है ऐसा क्यों हुआ?

- आपने/उन्होंने इसके बारे में क्या किया?

• मोनी की तरह आपकी उम्र के युवा किन स्थितियों में दुखी या बहुत चिंतित महसूस करते हैं?

• आपको क्या लगता है कि ऐसा होने पर वे क्या करते हैं?

**मदद मांगना**

• आपकी राय में, मोनी जैसे उन युवाओं की मदद के लिए क्या किया जा सकता है जो दुःख महसूस कर रहे हैं या बहुत ज्यादा चिंता कर रहे हैं?

• अगर मोनी जैसे युवाओं की मदद के लिए परामर्श /बातचीत होती, तो आपको क्या लगता है ये सुबिधा देने के लिए किस पर भरोसा किया जाएगा? (जांच: एक परामर्शदाता (एए फ एचसी), प्रशिक्षण प्राप्त एक पुराना सहकर्मी, कोई और?)

• उक्त सेवा कहां देना चाहिए ताकि यह उन युवाओं तक पहुंच सके जिनको इसकी आवश्यकता है? (जांच: स्कूल, क्लिनिक, सामुदायिक स्थल, कोई अन्य स्थान?)

• ऐसी कौन सी चीजें हैं जो युवाओं को इस मदद के लिए आने के लिए प्रोत्साहित करेंगी या उन्हें आने से रोकेंगी? (जांच: कुछ और?)

• आप किसी मित्र को सेवा जाने के लिए तक कैसे प्रोत्साहित कर के क्या कहेंगे?

**समापन**

• क्या आप कुछ और कहना चाहते हैं जिसके बारे में युवा लोग दुखी या चिंतित हैं, और उनकी मदद के लिए क्या किया जा सकता है?

• क्या आपके पास मेरे लिए कोई सवाल है?

हमें अपना समय और अपने विचार देने के लिए बहुत-बहुत धन्यवाद।
