# Supplementary material for: Concepts of psychosocial distress and help-seeking preferences among Indigenous adolescents: A qualitative study from Jharkhand, India
Source: PLOS Ment Health. 2026 May 4;3(5):e0000492. doi: 10.1371/journal.pmen.0000492 (PMC13138629; doi:10.1371/journal.pmen.0000492)
Supplement: S2 Text — (DOCX) [file pmen.0000492.s002.docx]

**S2 Text. Consolidated Criteria for Reporting Qualitative Studies (COREQ)**

| Item No | Guide Questions/Description | Reported on Page # |  | |
| --- | --- | --- | --- | --- |
| Domain 1: Research team and reflexivity | | |  | |
| Personal Characteristics | | |  | |
| 1. Interviewer/ facilitator | Which author/s conducted the interview or focus group? | 4 |  | |
| 2. Credentials | What were the researcher’s credentials? E.g., PhD, MD | 4 |  | |
| 3. Occupation | What was their occupation at the time of the study? | 4 |  | |
| 4. Gender | Was the researcher male or female? | 4 |  | |
| 5. Experience and training | What experience or training did the researcher have? | 4 |  | |
| Relationship with participants | | |  | |
| 6. Relationship established | Was a relationship established prior to study commencement? | 5-6 |  | |
| 7. Participant knowledge of the interviewer | What did the participants know about the researcher? e.g. personal goals, reasons for doing the research? | 5-6 |  | |
| 8. Interviewer characteristics | What characteristics were reported about the interviewer/facilitator? e.g. Bias, assumptions, reasons and interests in the research topic | 5-6 |  | |
| Domain 2: study design | | |  | |
| Theoretical framework | | |  | |
| 9. Methodological orientation and Theory | What methodological orientation was stated to underpin the study? e.g. grounded theory, discourse analysis, ethnography, phenomenology, content analysis | 4 |  | |
| Participant selection | | |  | |
| 10. Sampling | How were participants selected? e.g., purposive, convenience, consecutive, snowball | 4 |  | |
| 11. Method of approach | How were participants approached? e.g., face-to-face, telephone, mail, email | 5 |  | |
| 12. Sample size | How many participants were in the study? | 5 |  | |
| 13. Non-participation Setting | How many people refused to participate or dropped out? Reasons? | 5 |  | |
| 14. Setting of data collection | Where was the data collected? e.g., home, clinic, workplace | 5 |  | |
| 15. Presence of nonparticipants | Was anyone else present besides the participants and researchers? | No, 5 |  | |
| 16. Description of sample | What are the important characteristics of the sample? e.g. demographic data, date | S1 Table |  | |
| Data collection | | |  |  |
| 17. Interview guide | Were questions, prompts, and guides provided by the authors? Was it pilot tested? | S1 Text |  | |
| 18. Repeat interviews | Were repeat interviews carried out? If yes, how many? | No |  | |
| 19. Audio/visual recording | Did the research use audio or visual recording to collect the data? | Audio only, 5 |  | |
| 20. Field notes | Were field notes made during and/or after the interview or focus group? | Yes, 5 |  | |
| 21. Duration | What was the duration of the interviews or focus group? | 5 |  | |
| 22. Data saturation | Was data saturation discussed? | 5-6 |  | |
| 23. Transcripts returned | Were transcripts returned to participants for comment and/or correction? | No |  | |
| Domain 3: analysis and findings | | |  | |
| Data analysis | | |  | |
| 24. Number of data coders | How many data coders coded the data? | 5 |  | |
| 25. Description of the coding tree | Did the authors provide a description of the coding tree? | 6 |  | |
| 26. Derivation of themes | Were themes identified in advance or derived from the data? | 6 |  | |
| 27. Software | What software, if applicable, was used to manage the data? | 5 |  | |
| 28. Participant checking | Did participants provide feedback on the findings? |  |  | |
| Reporting | | |  | |
| 29. Quotations presented | Were participant quotations presented to illustrate the themes/findings? Was each quotation identified? e.g., participant number | 6-8 |  | |
| 30. Data and findings consistent | Was there consistency between the data presented and the findings? | 6-8 |  | |
| 31. Clarity of major themes | Were major themes clearly presented in the findings? | 6-8 |  | |
| 32. Clarity of minor themes | Is there a description of diverse cases or a discussion of minor themes? | 6-9 |  | |
