# Supplementary material for: Concepts of psychosocial distress and help-seeking preferences among Indigenous adolescents: A qualitative study from Jharkhand, India
Source: PLOS Ment Health. 2026 May 4;3(5):e0000492. doi: 10.1371/journal.pmen.0000492 (PMC13138629; doi:10.1371/journal.pmen.0000492)
Supplement: S1 Table — (DOCX) [file pmen.0000492.s004.docx]

**S1 Table. Qualitative methods used for each participant group**

| Data collection mode | 10-14 Girls | 10-14 Boys | 15-19 Girls | 15-19 Boys | Teachers | Parents | Health  Workers | Ekjut Staff (Peer Facilitators) | Total |
| --- | --- | --- | --- | --- | --- | --- | --- | --- | --- |
| Semi-structured Interviews: married adolescents | 1 | 1 | 9 | 6 |  |  |  |  | 16 |
| Semi-structured Interviews: married adolescents |  |  | 1 | 1 |  |  |  |  | 2 |
| Small Group Discussions* | 2 |  |  | 1 |  |  |  |  | 4 |
| Semi-structured Interviews: Health Workers |  |  |  |  |  |  | 2 |  | 2 |
| Semi-structured Interviews: Ekjut Staff |  |  |  |  |  |  |  | 1 | 1 |
| Focus Group Discussions ** | 1 | 1 | 1 |  | 2 | 1 |  |  | 5 |
| Total | 3 | 2 | 11 | 8 | 2 | 1 | 2 | 1 | 30 |

* Small group discussion: 3-5 participants

** Focus group discussion: 6-10 participants
