# Supplementary material for: Concepts of psychosocial distress and help-seeking preferences among Indigenous adolescents: A qualitative study from Jharkhand, India
Source: PLOS Ment Health. 2026 May 4;3(5):e0000492. doi: 10.1371/journal.pmen.0000492 (PMC13138629; doi:10.1371/journal.pmen.0000492)
Supplement: Attachment — Submitted filename: PLMH_MADAD_RESPONSE TO EDITORS AND REVIEWERS_R1_14Mar2026.docx [file pmen.0000492.s007.docx]

**RESPONSE TO REVIEWERS**

PMEN-D-25-00421R1

Concepts of psychosocial distress and help-seeking preferences among Indigenous adolescents: a qualitative study from Jharkhand, India

We thank the editors and reviewers for very helpful comments.

We have made the following changes in response.

| 1 | EDITORIAL COMMENTS | RESPONSE |
| --- | --- | --- |
| 1.1 | Please include a complete copy of PLOS’ questionnaire on inclusivity in global research in your revised manuscript. […] Please upload a completed version of your questionnaire as Supporting Information when you resubmit your manuscript. | We have now attached a complete copy of PLOS’ questionnaire on inclusivity in global research as Supporting Information. |
| 1.2 | Please amend your detailed Financial Disclosure statement. This is published with the article. It must therefore be completed in full sentences and contain the exact wording you wish to be published. | We have now edited the detailed Financial Disclosure statement. |
| 1.3 | Please ensure that your Ethics Statement is available in its entirety at the beginning of your Methods section, under a subheading 'Ethics Statement'. | We have moved the Ethics statement to the beginning of the Methods section (pp.4-5) and labelled it as such. |
| 1.4 | If there are ethical or legal restrictions on sharing a de-identified data set, please explain them in detail (e.g., data contain potentially identifying or sensitive patient information, data are owned by a third-party organization, etc.) and who has imposed them (e.g., a Research Ethics Committee or Institutional Review Board, etc.). Please also provide contact information for a data access committee, ethics committee, or other institutional body to which data requests may be sent. | The qualitative transcripts contain potentially identifying participant data. In addition, some data were transcribed by hand and transcripts only exist in paper form and are harder to share and anonymise.  We are sharing the final framework matrix as a minimal anonymised dataset through the UCL Discovery repository (ID: 946794E5-8312-411E-85F9-14641FFD0434).  We have updated the Data Availability statement in the submission form accordingly. |
|  | Reviewer 1 - comments |  |
| 2.1 | Along with positionality, the authors could summarise the measures taken for rigor and trustworthiness. | Thank you for this important suggestion.  We have now added a section on Measures to increase trustworthiness after the positionality statement on p.10.  This reads:  **“**Our overall approach to increase trustworthiness built upon the four principles summarised by Ahmed [29]: credibility achieved through extended involvement with concerned communities by Ekjut team members, discussions about personal biases and preconceptions throughout the research process, and triangulation of results between participant groups; a critical assessment of transferability drawing upon detailed descriptions of the communities’ characteristics and clear description of the sampling process; dependability, which we achieved through an audit trail linked to the development of the Framework matrix; and confirmability, as established through peer debriefing. “ |
| 2.2 | Consider using COREQ checklist while reporting, though most of the points are covered, some aspects are to be addressed. | Thank you, we used and included the COREQ checklist in the supplementary files and have now explained this on p.10. |
| 2.3 | Characteristics of participants could be summarized in the beginning of results | Thank you, we have moved these details to the first paragraph of the results section on p.10. |
| 2.4 | The authors mention that they use framework matrix analysis but I could not see it in the results. It looked like only a thematic analysis. To justify or clarify how the analysis fits the framework matrix analysis. I could not see multi-case comparisons. | Thank you, we have uploaded the Framework Matrix as a minimal dataset through the UCL Discovery repository (ID: 946794E5-8312-411E-85F9-14641FFD0434), and added reference and link to it on p.9. We have also clarified at what stage cross-case comparison occurred on p.9. The full matrix is too large to show as a table within the paper itself. |
| 2.5 | Add strengths and limitation of the study | We had included this as the central paragraph on p.2. |
| 2.6 | Line 71: “c.9%” there is a typing error | We have replaced (c. for circa) with ‘around’ on p.3. |
|  | Reviewer 2 - Comments |  |
|  | The methodological orientation can come before the study participants. | We have moved this section as requested (pp.6-7) |
| 3.1 | While the richness of the qualitative data is a strength, the Results section would benefit from greater analytic condensation. Several quotations illustrate similar points, and selective reduction of excerpts particularly where meanings overlap could improve clarity and strengthen the analytic narrative without loss of nuance. | Thank you, we have edited the results section throughout to improve clarity. These changes are highlighted in the revised manuscripts on pp.10-20. |
| 3.2 | The concept of “udu” emerges as a central idiom of distress in this study. However, the Discussion could further strengthen the manuscript by more explicitly articulating how “udu” advances existing idioms-of-distress literature (e.g., in relation to “tension” or “thinking too much”) and what this implies for assessment, engagement, or intervention design in adolescent mental health programmes. The manuscript would benefit from a more focused discussion of how the findings could inform the design of culturally grounded, community-based adolescent mental health interventions, particularly in Indigenous or rural contexts where access to formal services is limited. | Thank you. We have discussed the relationship between ‘udu’, ‘tension’ and ‘thinking too much’ in the first paragraph of p.21.  We have added more discussion on how findings could inform the design of culturally grounded, community-based adolescent mental health interventions in Indigenous or rural contexts where access to formal services is limited on p.22. |
| 3.3 | Framework analysis is appropriate, but some procedural details are brief. The authors may consider adding brief clarification on how coding disagreements were resolved and how saturation was operationalised in practice, to further strengthen transparency and methodological rigor. | We described our approach to saturation on p.8, including with an example. We described the stage at which coding uncertainties (rather than disagreements) were resolved and how on p.9. |
